# Supplementary material for: Vegetation structural change since 1981 significantly enhanced the terrestrial carbon sink
Source: Nat Commun. 2019 Sep 18;10:4259. doi: 10.1038/s41467-019-12257-8 (PMC6751163; doi:10.1038/s41467-019-12257-8)
Supplement: Supplementary file 1 — Supplementary Information [file 41467_2019_12257_MOESM1_ESM.pdf]

Supplementary Information for

Vegetation Structural Change Since 1981 Significantly Enhanced the Terrestrial Carbon Sink

Jing M. Chen[1,2], Weimin Ju[*2,3], Philippe Ciais, Nicolas Viovy[4], Ronggao Liu[5], Yang Liu[5], Xuehe Lu[2]

[*] Corresponding author: juweimin@nju.edu.cn

August 28, 2019

[*] Correspond to: juweimin@nju.edu.cn

1. Supplementary Figures

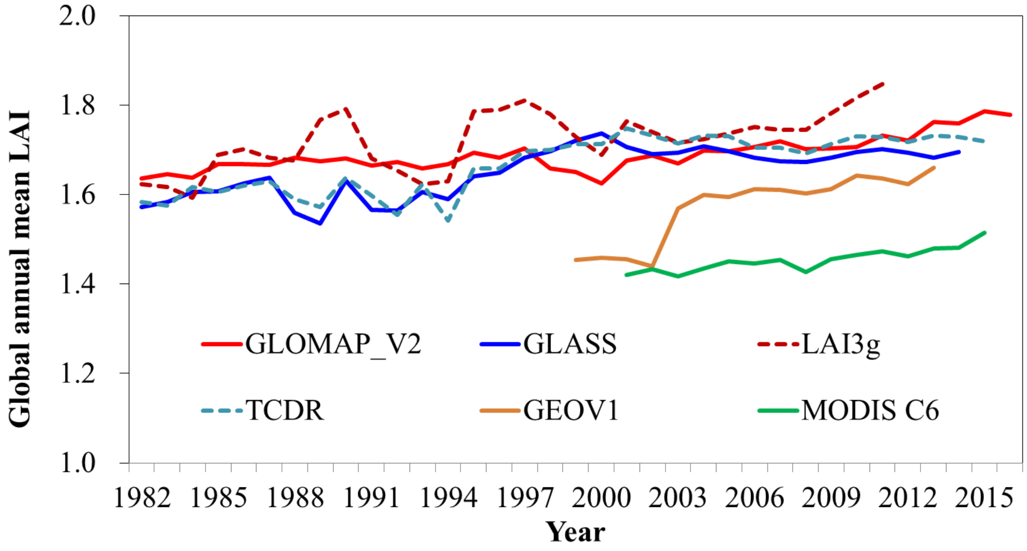

Supplementary Figure 1*. Comparison of LAI products for their annual mean values during the growing season defined as months with average air temperature greater than zero. Among 4 long-term LAI products, GLOBMAP_V2, GLASS and LAI3g are used in this study, while TCDR is not used for its similarity with GLASS. The trend and uncertainty of each time series is shown in Supplementary Table 1. This set of LAI products is much improved from that shown in Jiang et al. (2017), in which GLOBMAP_V1 and GLASS were affected by MODIS C5 reflectance data which caused negative trends in LAI after 2000. As this issue is overcome here, all products show significant increasing trends.*

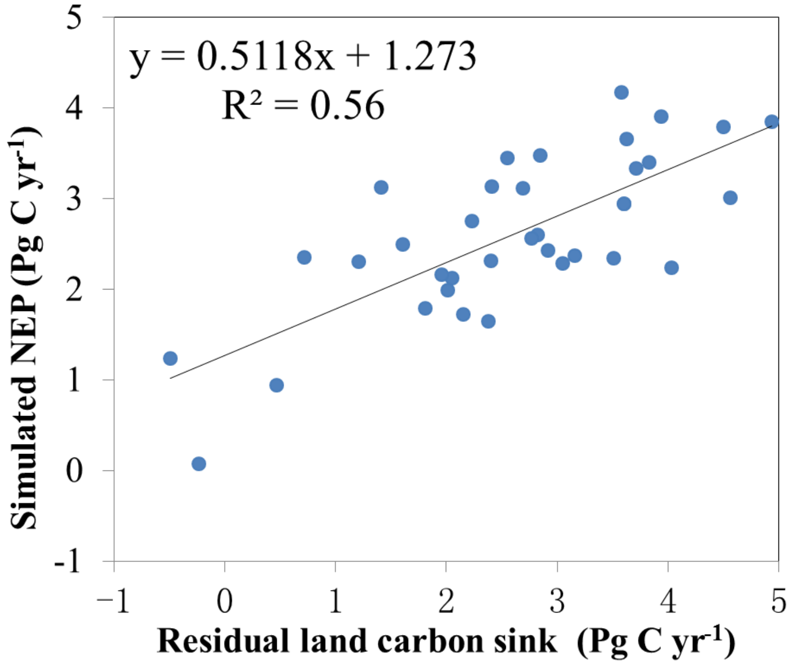

Supplementary Figure 2*. Comparison of simulated global NEP with the residual land sink computed as the sum of fossil fuel, cement, and land use change minus the sum of atmospheric CO_2 growth rate and ocean sink.*

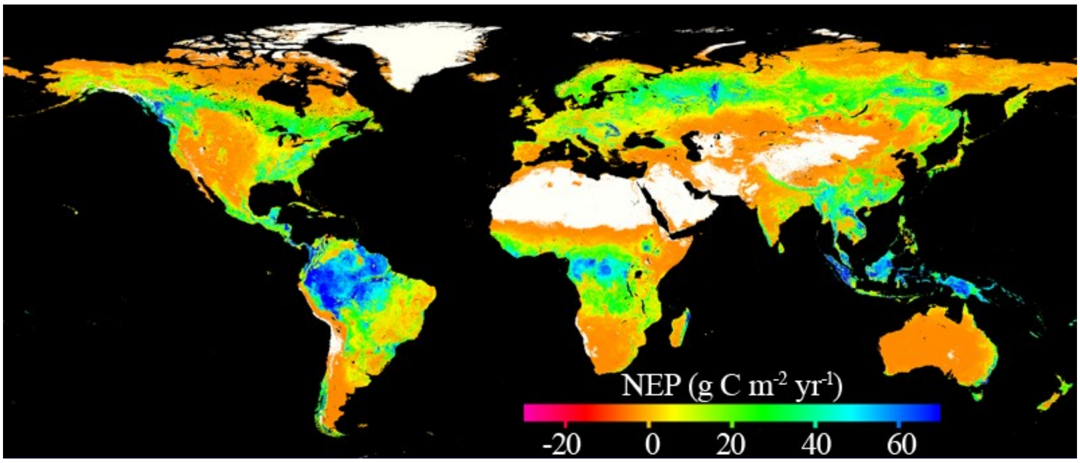

Supplementary Figure 3. *The average legacy effect on the land carbon sink over the period from 1981 to 2016 due to changes prior to 1981 in the various drivers including CO_2 fertilization, climate and nitrogen deposition. The effect is all positive, although non-vegetated areas (white) are excluded.*

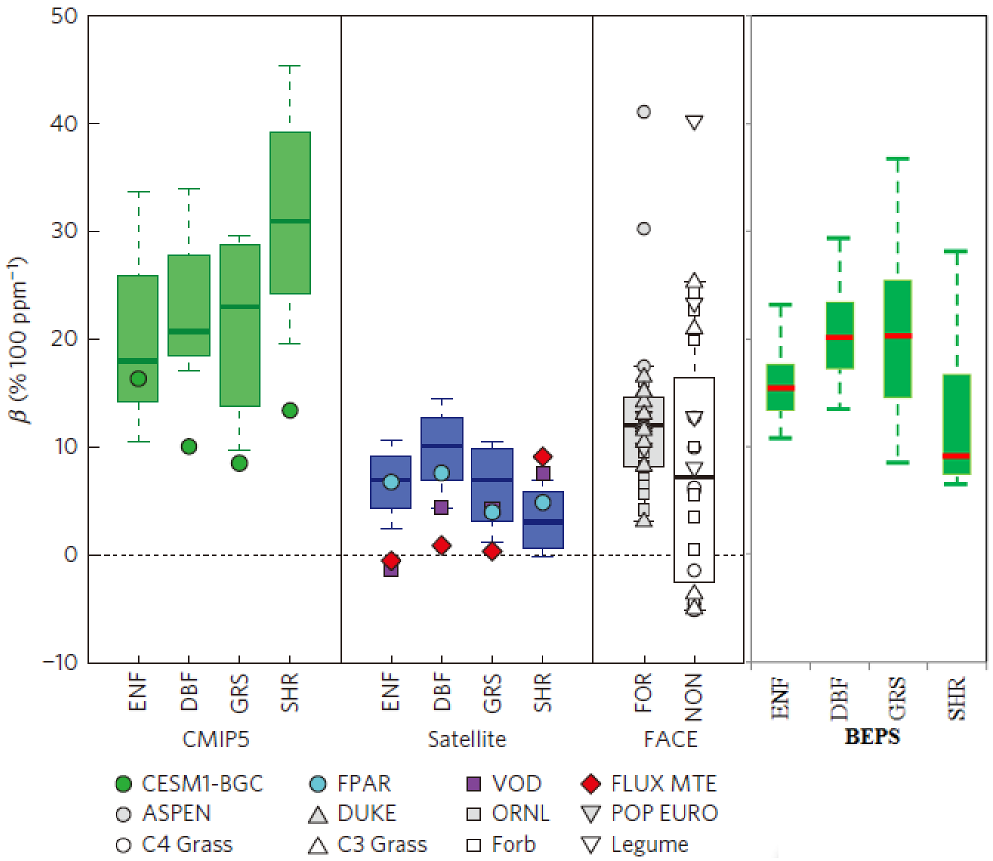

Supplementary Figure 4. *The sensitivity of NPP to atmospheric CO_2 concentration for evergreen needleleaf forest (ENF), deciduous broadleaf forest (DBF), grass (GRS) and shrub (SHR). The β factor is defined as the change in NPP per 100 ppm increase in atmospheric CO_2 concentration. The left and middle plots were adopted from Smith et al. (2016) with the right plot added showing results from the BEPS model. The β factors of BEPS were the averages of individual pixels for different cover types. The bottom and top of the rectangle indicate the first and third quartiles. The horizontal line within a rectangle represent the median value. The error bars above the top and below the bottom of the rectangle indicate the maximum and minimum values, respectively.*

The overall β value in the northern hemisphere simulated by the BEPS model was 18.6% over the period from 1981 to 2016. The CO_2 fertilization effect, i.e. the β factor, is calculated as:

$$\left[\frac{NPP_{CO2,2016} - NPP_{baseline,2016}}{NPP_{baseline,2016}} \times \frac{100}{CO_{2,2016} - co_{2,1981}}\right] \times 100\%$$

where $NPP_{CO2,2016}$ and $NPP_{baseline,\ 2016}$ are, respectively, the NPP values simulated under the scenario of CO_2 change only (Simulation II) and the baseline in year 2016. $CO_{2,2016}$ and $CO_{2,1981}$ are the CO_2 concentration in 2016 and 1981, respectively. The β values obtained in this study are compared with those in Smith et al. (2016) for four major plant functional types including evergreen needleleaf forest (ENF), deciduous broadleaf forest (DBF), grass (GRS), and shrub (SHR) (Supplementary Figure 4).

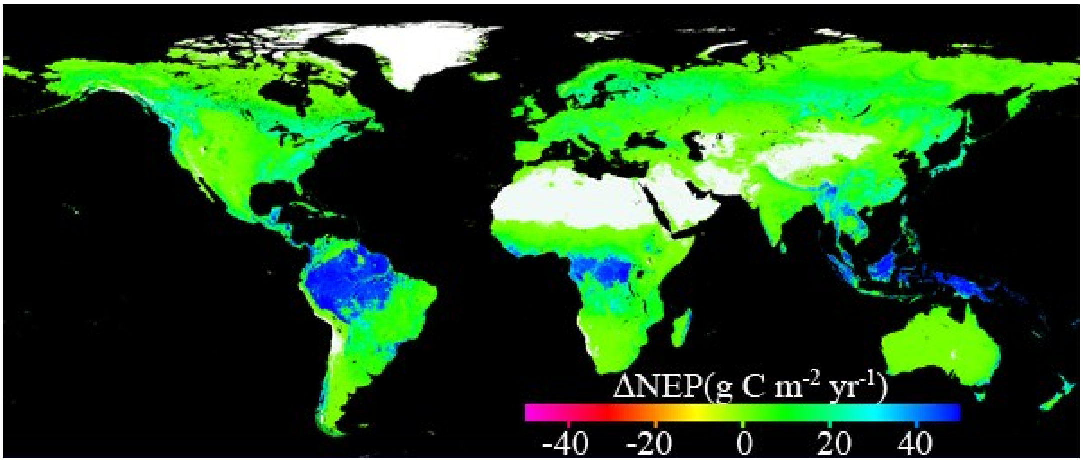

Supplementary Figure 5. *Average C sink enhancement due to CO_2 concentration change over the period from 1981 to 2016. Values shown are means of annual NEP simulated from Simulation II (only CO_2 concentration changes historically while other factors remained temporally unchanged) minus that the output from Simulation I (all factors remained temporally unchanged) for the study period. Positive values indicate that CO_2 concentration change enhanced C sequestration, vice versa. The contributions of different drivers to the enhancement of the terrestrial carbon sink were quantified according to differences between NEP simulated in individual simulations minus the value in the baseline simulation. The difference in NEP between Simulation II and Simulation I can then be attributed to the sink enhancement due to CO_2 fertilization over the baseline. The carbon sink enhancements by changes in vegetation structure, nitrogen deposition, and climate are shown in Supplementary Figures 6, 8, and 9. In Simulation V, only climate data are historically variable for the purpose of isolating its effect on the land carbon sink (Supplementary Figure 9). In Simulation VI, historical datasets of LAI, climate, nitrogen deposition, and CO_2 concentration were used to drive the BEPS model. The difference in NEP between Simulation VI and Simulation I determines the integrated sink enhancement due to all drivers considered (Supplementary Figure 10).*

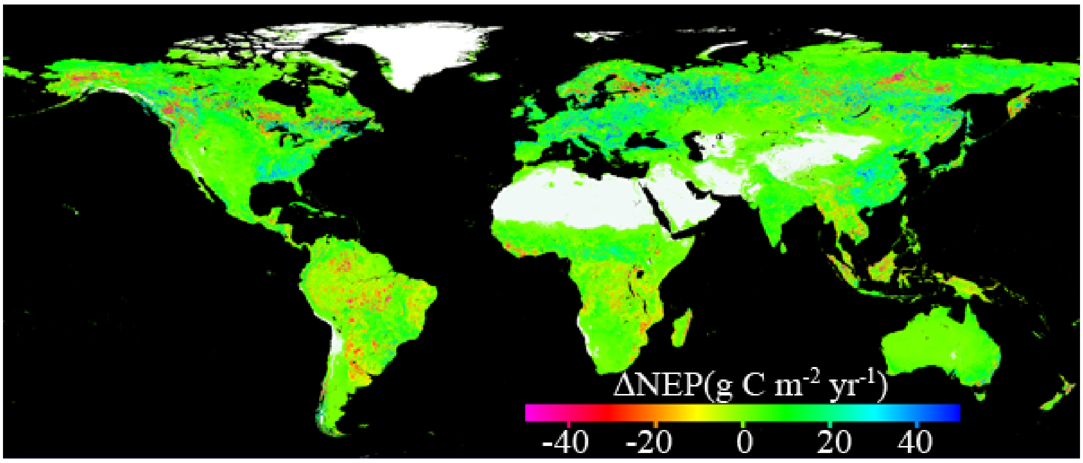

Supplementary Figure 6. *Average C sink enhancement/reduction due to LAI change over the period from 1981 to 2016. Values shown are the means of annual NEP simulated from Simulation III (only LAI changed temporally and other factors remained temporally unchanged during 1981-2016) minus that the output from Simulation I (all factors remained temporally unchanged) for the study period. Positive values indicate that LAI change enhanced the C sink, and vice versa.*

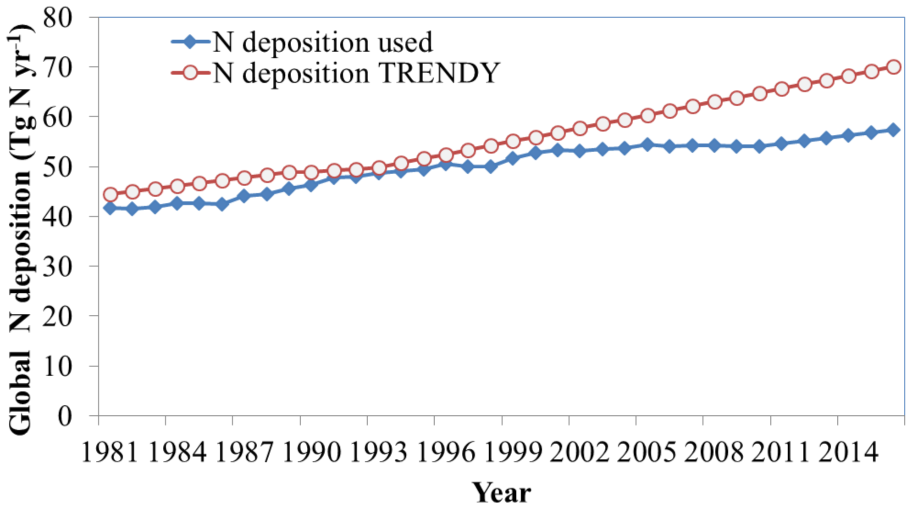

***Supplementary Figure** 7. Comparison of nitrogen deposition datasets used in this study and by TRENDY models. These two datasets are similar before 1990 as both are based on measurements, but the increasing trends after 1990 are different because the data used by the models are based on linear extrapolation from 1990 to 2050, at which the nitrogen deposition is estimated based on projected anthropogenic sources and other assumptions (Dentener, 2006), while satellite measurements from 2000-2009 are used in our dataset (Lu et al., 2014) and could follow the realistic trend more closely than the linearly extrapolated trend used by the models. Over the 1981-2016 period, the total nitrogen deposition is 301 Tg N in our study, while it is 403 Tg N in TRENDY. The difference could be due to the overall decrease in nitrogen deposition in North America and other regions in this period.*

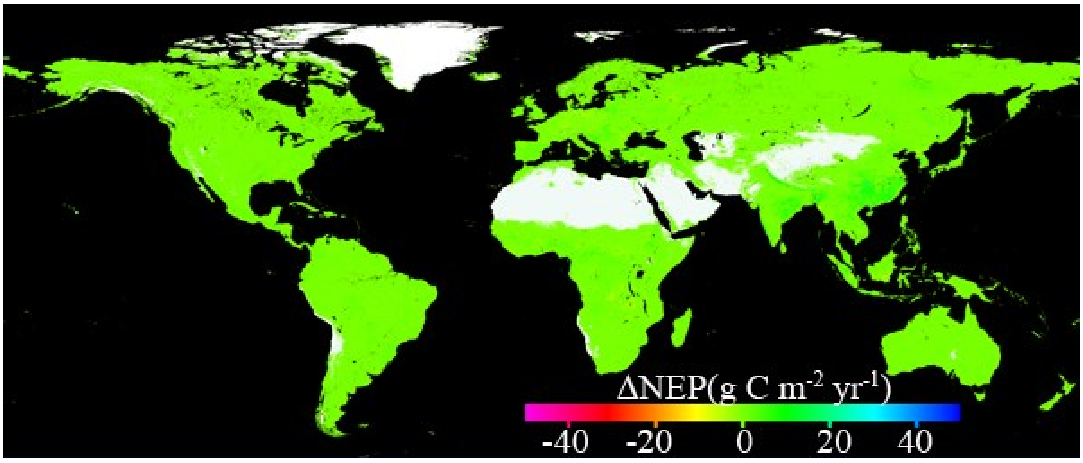

***Supplementary Figure 8.** Average C sink enhancement/reduction due to nitrogen deposition change over the period from 1981 to 2016. Values shown are means of annual NEP simulated from Simulation IV (only nitrogen deposition temporally changed while other factors remained temporally unchanged) minus that the output from Simulation I (all factors remained temporally unchanged) for the study period. Positive values indicate that nitrogen deposition enhanced the C sink, and vice versa.*

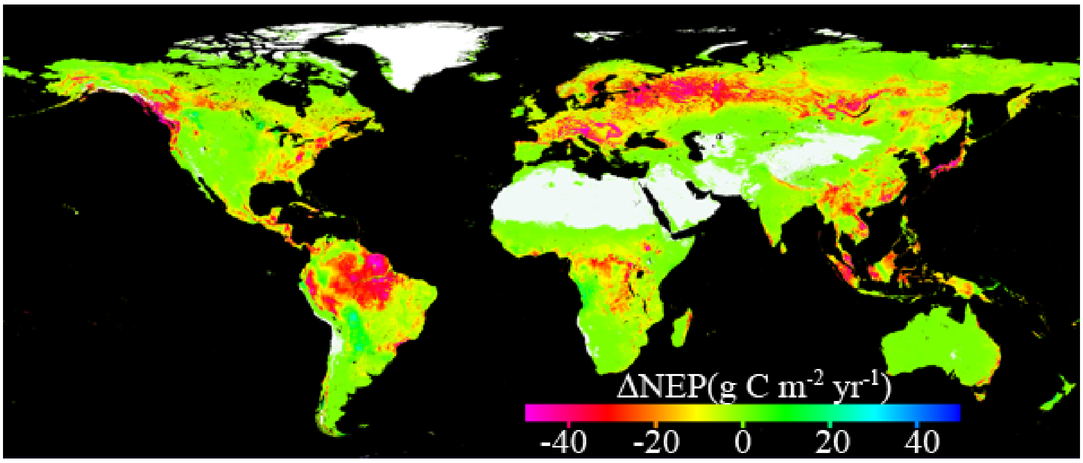

Supplementary Figure 9. *Average C sink enhancement/reduction due to climatic change over the period from 1981 to 2016. Values shown are the means of annual NEP simulated from Simulation V (only climate followed historical variations while other factors remained temporally unchanged) minus that the output from Simulation I (all factors remained temporally unchanged) for the study period. Positive values indicate that climate change enhanced the C sink, and vice versa.*

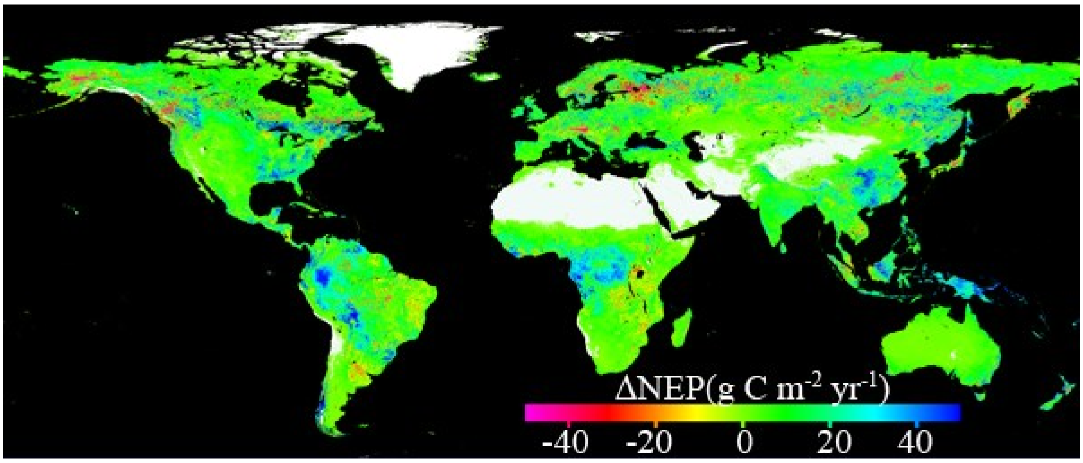

Supplementary Figure 10. *Average C sequestration enhancement due to the integrated effect of CO_2 concentration, nitrogen deposition, LAI, and climate changes over the period from 1981 to 2016. Values shown are means of the annual NEP output from Simulation VI (all factors vary temporally) minus that output from Simulation I (all factors remained unchanged) for the study period. Positive values indicate that the integrated effects of climate, LAI, nitrogen, and CO_2 concentration changes enhanced C sequestration, vice versa.*

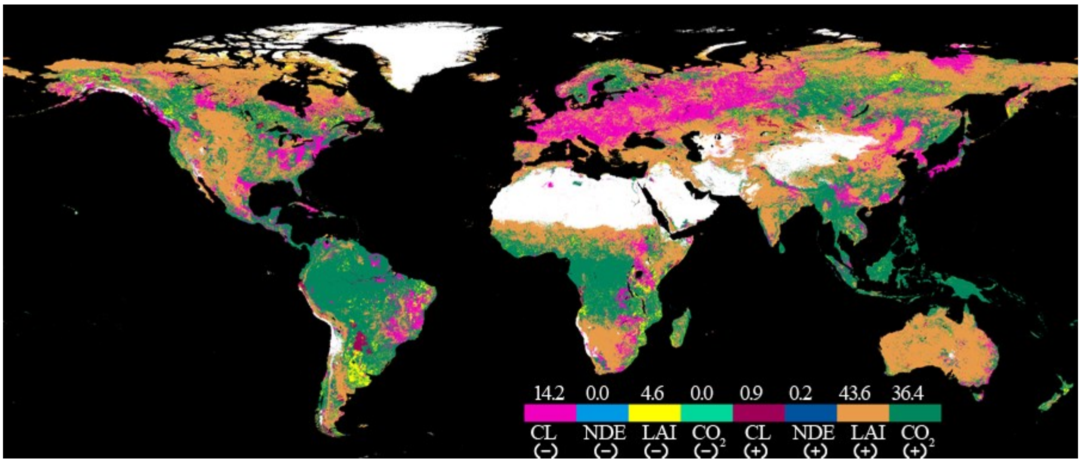

Supplementary Figure 11*. Spatial distribution of dominant drivers on the accumulated NEP during 1982 to 2016. At a location, the dominant driver is determined as the one causing the largest impact on the accumulated NEP among all drivers including climate (CL), nitrogen deposition (NDE), leaf area index (LAI), and CO_2. The postscript '-' of the drivers indicates a negative effect on accumulated NEP, whereas '+' indicates a positive effect. The value above each driver indicates the percentage of the affected area relative the global total vegetated area.*

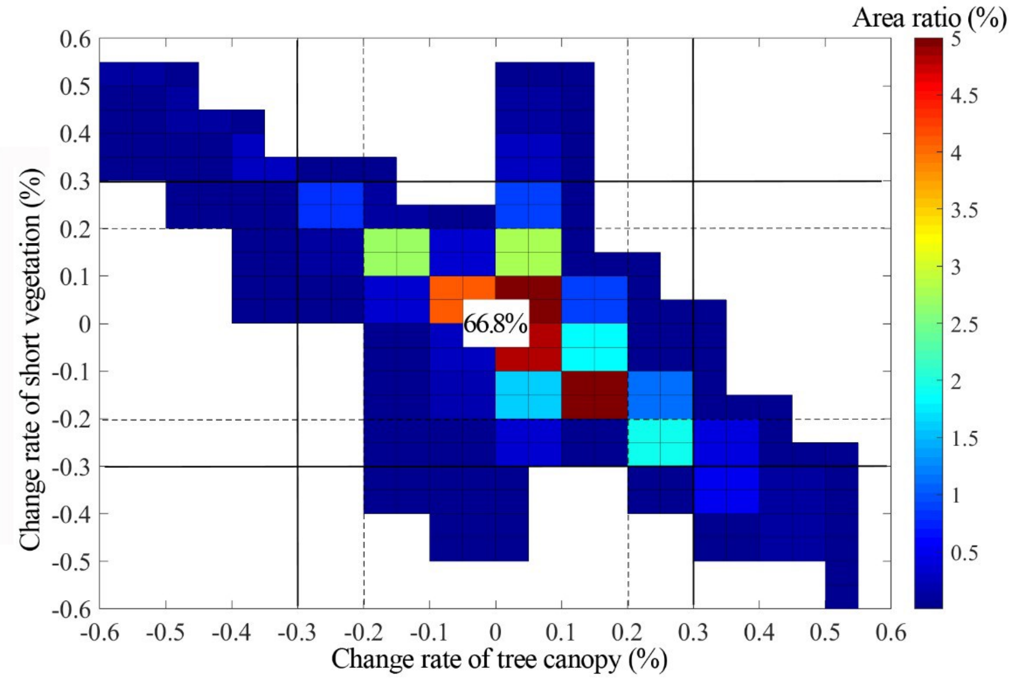

Supplementary Figure 12. *Areal percentages of landmass corresponding to given changing ratios of tree canopy (TC) and short vegetation (SV) coverage to the global total vegetative area. The number in the center indicates that 66.8% of the global total vegetative area did not experience any changes in tree canopy and short vegetation. The dashed and solid lines indicate the ±20% and ±30% changes in TC and SV, respectively. Disturbance (i. e. land cover change) affects carbon sequestration of terrestrial ecosystems. With Landsat data at 30 m resolution, Song et al. (2018) produced global land cover change maps, including short vegetation (SV), tree canopy (TC), and bare ground (BG) change maps over the period from 1982 to 2016. In 66.8% of the global vegetative areas, no changes of TC and SV occurred. Pixels with 20% and 30% increases or decreases in SV or TC account only 6.7% and 2.0% of the global total of vegetative land areas. Changes from SV to TC or from bare land to SV or TC may incur terrestrial carbon sinks (Supplementary Figures 13) which are associated with LAI changes (Supplementary Figure 14).*

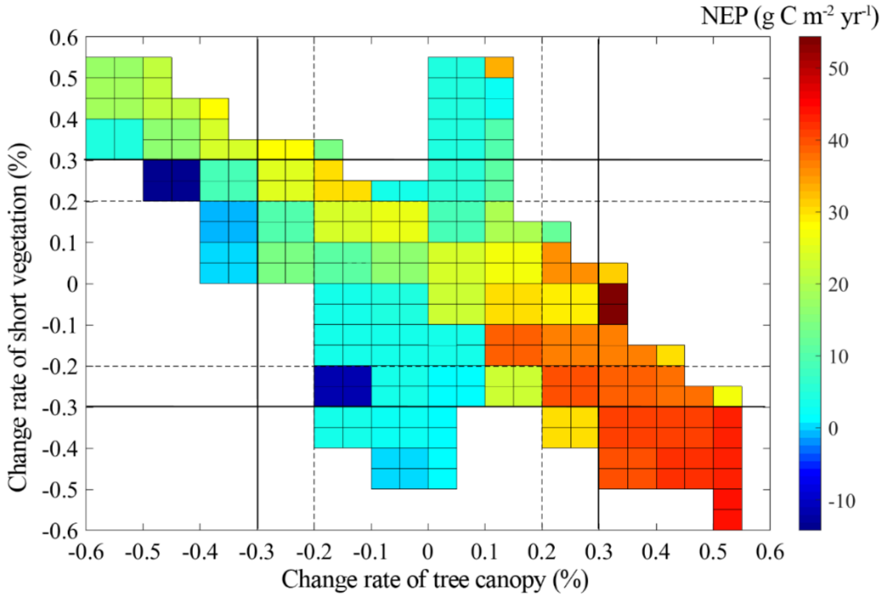

Supplementary Figure 13. *The impact of tree canopy and short vegetation coverage on the temporal trend of LAI during 1982-2016. Increases in tree canopy coverage are all associated with increasing LAI, while increases in short vegetation coverage can induce either increase or decrease in LAI depending on whether they replace tree canopies or bare land. The dashed and solid lines indicate the ±20% and ±30% changes in TC and SV, respectively.*

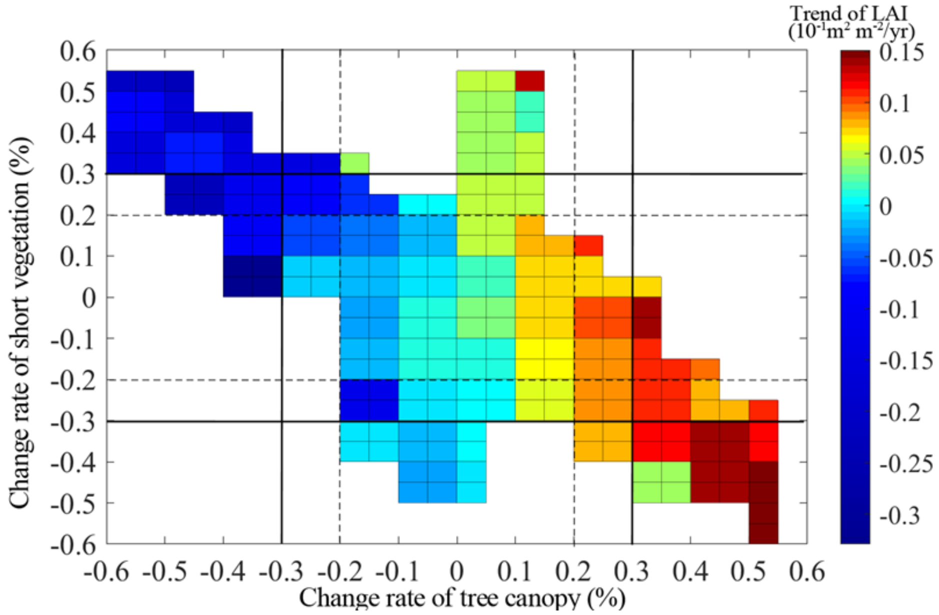

Supplementary Figure 14. *The impact of tree canopy and short vegetation coverage on NEP averaged during 1982-2016. Increases in tree canopy coverage are associated with decreases in short vegetation coverage and sinks, while increases in short vegetation could be either sources or sinks depending on whether they replace tree canopies or bare land. The dashed and solid lines indicate the ±20% and ±30% changes in TC and SV, respectively.*

2. Supplementary Tables

Supplementary Table 1. *Trends of global annual mean LAI (m^2 m^{-2} yr^1) of different LAI products*

Data source	Trends of global annual mean LAI						
	GLOBMAP 1982-2016	GLASS 1982-2015	TCDR 1982-2015	LAI3g 1982-2011	GLOBMAP 2001-2016	MODIS 2001-2016	GEOV1 1999-2013
AVHRR+ MODIS	**0.0031***** (0.00039)						
AVHRR		**0.0042***** **(0.00063)**	**0.0052***** (0.00062)	**0.0049**** (0.00108)			
MODIS					**0.0068***** (0.00078)	**0.0052***** (0.00074)	
SPOT/VGT							**0.0154***** (0.0022)

Note: Values in brackets are uncertainties; *** *and* ** *indicate the significance levels of 0.001 and 0.01, respectively.*

Supplementary Table 2. *Comparison of annual global terrestrial carbon sink simulated by different models again GCP residual carbon sink during 1981 to 2016*

Models	R^2	RMSE (Pg C yr^1)	Accumulated (Pg C)	Trend (Pg C yr^{-2})
CABLE	0.63	1.55	142.67	0.051
CLASS-CTEM	0.3	1.49	109.42	0.041
CLM4.5BGC	0.17	1.21	83.21	0.008
DLEM	0.35	1.04	85.91	0.042
ISAM	0.55	0.90	83.01	0.016
JSBACH	0.05	1.47	109.42	0.032
JULES	0.42	1.08	95.39	0.032
LPJ-GUESS	0.43	1.36	73.88	0.029
LPJ	0.65	1.02	70.25	0.046
LPX-Bern	0.3	1.88	43.11	0.017
OCNv2	0.56	0.99	113.27	0.041
ORCHIDEE	0.25	1.23	91.32	0.020
ORCHIDEE-MICT	0.29	1.14	106.22	0.020
SDGVM	0.23	1.30	73.62	0.045
VISIT	0.52	1.03	110.52	0.059
**BEPS**	**0.56**	**0.84**	**94.77**	**0.045**
GCP Residual carbon sink			94.32	0.059

Supplementary Table 3. *Definition of simulations conducted in the study*

Simulations	LAI	Climate	Nitrogen deposition	CO_2
I	1982-1986 average	Randomly sampled 1970-1979 climatology data.	Value in 1981	Value in 1981
II	1982-1986 average	Randomly sampled 1970-1979 climatology data.	Value in 1981	Changing value from 1981-2016
III	Changing value from 1981 to 2016	Randomly sampled 1970-1979 climatology data	Value in 1981	Value in 1981
IV	1982-1986 average	Randomly sampled 1970-1979 climatology data	Changing value from 1981-2016	Value in 1981
V	1982-1986 average	Changing climate from 1981 to 2016	Value in 1981	Value in 1981
VI	Changing value from 1981-2016	Changing climate from 1981 to 2016	Changing value from 1981-2016	Changing value from 1981-2016

Explanation of Table 3: In order to separate the effects of LAI, climate, nitrogen deposition, and CO_2 concentration on the carbon exchange during the period from 1981 to 2016, six simulations are conducted (Supplementary Table 3) for the1981- 2016 period. In Simulation I (the baseline), the BEPS model is driven by the average LAI during 1982-1986, nitrogen deposition and CO_2 concentration in 1981, and baseline climate data that are interannually variable with no temporal trend. These baseline climate data are created by replacing climate of each year during 1981-2016 with climate of a year randomly selected from 1970 to 1979. In Simulation II, the BEPS model is driven by the same datasets used in Simulation I with the exception of atmospheric CO_2 concentration, that follows the historical variation during 1981-2016 as measured in Mauna Loa. Simulation III used the same datasets as Simulation I except for LAI, which is the remotely sensed LAI time series, for the purpose of quantifying the carbon sink enhancement by vegetation structural change. In Simulation IV, only nitrogen deposition followed its historical records to investigate its effect on the land sink. In Simulation V, only climate data are historically variable for the purpose of isolating its effect on the land carbon sink. In Simulation VI, historical datasets of LAI, climate, nitrogen deposition, and CO_2 concentration were used to drive the BEPS model.

The residual land carbon sink of the Global Carbon Project is computed as the sum of fossil fuel, cement, and land use change emissions minus the sum of atmospheric and oceanic sinks (Le Quere

et al., 2018). The land use change emission is quantified as the difference between carbon emission during disturbance and carbon uptake by subsequent regrowth. In our study, the global NEP simulated by BEPS is considered as an approximation of the residual global land sink because remotely sensed LAI would be affected by both disturbance and regrowth. After disturbance, LAI first reduces abruptly and then increases slowly with regrowth. In the BEPS model, the abrupt reduction in LAI causes transfer of a portion of biomass carbon to soil organic matter which is then subsequently decomposed and emitted to the atmosphere, while the slow increase in LAI due to regrowth results in slow increase in carbon uptake. For one pixel in a given year, these two processes are not balanced, but in a landscape mosaic with various disturbance histories, it may be reasonable to assume that these two processes are balanced, making no net gain of carbon in the ecosystem. In other words, NEP simulated by BEPS using measured LAI data can be regarded as the residual land sink at regional and global scales if the change in the disturbance rate in a region is historically small. However, the disturbance rates in different regions are generally variable (Le Quere et al., 2018), making the simulated NEP differ from the residual land sink to some extent (Supplementary Figure 2). Compared with 15 prognostic models participated in GCP (Le Quere et al., 2018), BEPS results are mostly better in terms of the Pearson regression coefficient (R^2), root mean square error, accumulated total sink, and trend against the residual land sink reported by Le Quere et al (2018) (Supplementary Table 2).

3. Supplementary Discussion

The residual land carbon sink of the Global Carbon Project is computed as the sum of fossil fuel, cement, and land use change emissions minus the sum of atmospheric and oceanic sinks (Le Quere et al., 2018). The land use change emission is quantified as the difference between carbon emission during disturbance and carbon uptake by subsequent regrowth. In our study, the global NEP simulated by BEPS is considered as an approximation of the residual global land sink because remotely sensed LAI would be affected by both disturbance and regrowth. After disturbance, LAI first reduces abruptly and then increases slowly with regrowth. In the BEPS model, the abrupt reduction in LAI causes transfer of a portion of biomass carbon to soil organic matter which is then subsequently decomposed and emitted to the atmosphere, while the slow increase in LAI due to regrowth results in slow increase in carbon uptake. For one pixel in a given year, these two processes are not balanced, but in a landscape mosaic with various disturbance histories, it may be reasonable to assume that these two processes are balanced, making no net gain of carbon in the ecosystem. In

other words, NEP simulated by BEPS using measured LAI data can be regarded as the residual land sink at regional and global scales if the change in the disturbance rate in a region is historically small. However, the disturbance rates in different regions are generally variable (Le Quere et al., 2018), making the simulated NEP differ from the residual land sink to some extent (Supplementary Figure 2). Compared with 15 prognostic models participated in GCP (Le Quere et al., 2018), BEPS results are mostly better in terms of the Pearson regression coefficient (R^2), root mean square error, accumulated total sink, and trend against the residual land sink reported by Le Quere et al. (2018) (Supplementary Table 2).

Supplementary References

Dentener, F.J. Global maps of atmospheric nitrogen deposition, 1860, 1993, and 2050. Data set. Available on-line [http://daac.ornl.gov/] from Oak Ridge National Laboratory Distributed Active Archive Center, Oak Ridge, Tennessee, U.S.A. DOI:10.3334/ORNLDAAC/830 (2006)

Jiang, C.Y. et al. 2017. Inconsistencies of interannual variability and trends in long-term satellite leaf area index products. *Glob. Change Biol.* **23**, 4133-4146 (2017).

Le Quere, C. Global carbon budget 2017. *Earth Syst. Sci. Data* **10**, 405-448 (2018).

Lu, X.H. et al. Estimated global nitrogen deposition using NO_2 column density. *Int. J. Remote Sens.* **34**, 8893-8906 (2014).

Smith, W. K. et al. Large divergence of satellite and Earth system model estimates of global terrestrial CO_2 fertilization. *Nat. Clim. Change* **6**, 306-310 (2016).

Song, X.P. et al. Global land change from 1982 to 2016. *Nature* **560**, 639-643 (2018).
